# Relationship between dietary glycemic index and glycemic load and sperm-quality parameters in Iranian men: a cross-sectional study

**DOI:** 10.1186/s40795-024-00840-2

**Published:** 2024-02-26

**Authors:** Elham Hosseini, Mehdi Khodavandloo, Somaye Abdollahi Sabet, Seyedeh Neda Mousavi

**Affiliations:** 1 Zanjan Metabolic Diseases Research Center, Zanjan University of Medical Sciences, Zanjan, Iran; 2 Department of Community Medicine, School of Medicine, Zanjan University of Medical Sciences, Zanjan, Iran

**Keywords:** Diet, Glycemic index, Glycemic load, Sperm, Infertility

## Abstract

**Background:**

Infertility is a major clinical problem with psychological, financial and medical costs. Male infertility has recently been linked to 50% of childless couples. It is worth mentioning that diet and its components can be manipulated and applied in counseling meetings of infertile men as a modifiable factor. The goal of this study was to determine the correlation of dietary glycemic index (GI) and glycemic load (GL) with sperm-quality parameters in Iranian men.

**Materials and methods:**

In this cross-sectional study which was carried from Aug to Nov 2023, after excluding smokers, 322 men who were attending the IVF clinic of Ayatollah Mousavi Hospital for seminal analysis were enrolled. Dietary intake was completed by an expert dietitian through face-to-face interviews with a validated 168-item food frequency questionnaire (FFQ). In the present study, sperm-quality parameters, including motility, concentration, normal morphology, vitality, DNA fragmentation index (DFI), and chromatin maturation of sperm, were analyzed. The relationship between dietary GI and CL and these parameters was assessed.

**Results:**

Adjusting for the baseline variables, dietary GI and GL showed positive and negative associations with sperm progressive motility, respectively (*p* = 0.01 and *p* < 0.001). Higher dietary GI was associated with lower (*p* = 0.03); however, a higher dietary GL was associated with higher immotile sperm (*p* < 0.001). A higher dietary GI was associated with a 77% higher sperm count (*p* = 0.003). In contrast, higher dietary GL was associated with a lower count (*p* < 0.001). Higher dietary GI and GL were associated with higher and lower percentages of sperm with normal morphology by 70% and 40%, respectively (*p* < 0.001 in both). A higher dietary GL was associated with an increase in sperm vitality and DFI of 33% and 53%, respectively (*p* < 0.001). Higher dietary GI showed a significant negative effect on sperm DFI (*p* = 0.009). Dietary GI and GL showed significant positive and negative effects on SCMA, respectively (*p* = 0.002 and *p* < 0.001).

**Conclusion:**

A diet with a higher GI showed beneficial effects on more parameters of sperm; however, higher dietary GL showed deleterious effects, except sperm vitality and SCMA. More studies are needed to obtain a concise result.

## Background

Infertility, as the failure to achieve clinical pregnancy in the period of ≥ 12 months with regular unprotected sexual intercourse, is caused by disorders in the male and female reproductive systems and globally affects 20–30% of couples with an alarming increasing rate [1–3]. Sperm is a male sex cell that is produced from germ cells and finally converts to mature sperm over three months. The number, movement and shape of sperm play a significant role in male fertility. A recent decrease in the quality of seminal fluid has no correlation with genetics, disease, or hormones. It is interesting to note that the reason for male infertility in different countries has various reasons based on geographical regions, such as lifestyle, dietary habits and environmental factors, which show the urgent need to conduct studies to determine these triggers affecting this disorder [4, 5]. A decrease in the quality of sperm in seminal fluid is one of the main reasons for male infertility [6], including azoospermia, oligospermia, and abnormalities in sperm morphology, motility, volume and concentration [7, 8]. Several possible reasons are proposed for the decrease in semen quality, including environmental pollution, stress, anxiety, and unhealthy eating habits [9]. Recently, society’s lifestyle changed due to the nutritional transition from high physical activity per day and a hypo-caloric traditional diet with high intake of complex carbohydrates and fiber with low glycemic index and load (GI and GL) to sedentary lifestyle, hypercaloric diets with high intake of refined carbohydrates and low fiber with high GI and GL, which are negatively associated with infertility [3]. A properly balanced diet with a suitable quality and quantity can affect male and female fertility. Daily calorie intake from macronutrients, including carbohydrates, fat, and protein, as well as specific micronutrients, can affect fertility [10, 11]. Growing scientific evidence highlights the beneficial effects of a diet based on plant protein that is rich in antioxidants, fiber, and low-GI carbohydrates on fertility [10–12].. A diet with high GI and GL decreases fertility status by affecting insulin sensitivity, percentage of body fat, and leptin concentrations [13]. In a recent Iranian cross-sectional study, a significant inverse association was found between sperm quality parameters and BMI and waist circumference [14]. Another case-control study reported lower reproductive hormonal levels in participants with or without diabetes [15]. To our knowledge, there are limited Iranian studies on the association between diet and fertility. Despite the proven evidence on the correlation between dietary GI and load GL with some chronic diseases, including cardiovascular diseases, cancers and metabolic disorders [16, 17], this association has not been studied to date with sperm-quality parameters in Zanjan City, a mountainous region with a Turkish population and special dietary habits. Therefore, for the first time, the association between dietary GI and GL was studied with sperm-quality parameters, including count, motility, vitality, movement, DNA fragmentation index (DFI) of sperm and sperm chromatin maturation (SCMA), in Iranian men.

## Methods

### Participants and study design

In the present cross-sectional study which was done between 23 Aug to 20 Nov 2023, 400 adult men aged 20–50 year. who attended the IVF clinic of Ayatollah Mousavi Hospital, Zanjan, Iran, for sperm analysis were randomly selected. An informed assigned form was gathered from all participants. Men with a history of testicular atrophy, urinary tract infection, vasectomy, varicocele, azoospermia, testicular torsion, genital surgery, other genital diseases, endocrine, anatomical disorders, samples taken by a testicular sperm extraction (TESE) procedure, men with a history of metabolic diseases including cardiovascular disease, cancers, diabetes or impaired fasting glucose and impaired glucose tolerance, kidney disease or osteoporosis, psychiatric and physiological disorders such as depression, alcohol and drug abuse, intake of supplements, previous hormone therapy, anticoagulants, anti-androgens, androgens, cytotoxic drugs or immune suppressants were not included in the study. Athletes and men on a special diet were excluded. After dietary analysis, participants with calorie intake lower than 800 kcal or higher than 5000 kcal were excluded. Final analysis was performed on 322 men. The ethics committee of Zanjan University of Medical Sciences approved the present study (IR.ZUMS.REC.1402.128).

### Morphometric and social characterization

Anthropometric measures, including weight and height, were measured by a standard and calibrated scale. Weight was measured without shoes with the least cloths, and height was measured in a relaxed situation by a nonelastic meter on the wall while the shoulders, buttocks, heels and behind of the head were attached to the wall and the patient looked forward. Then, body mass index (BMI) was computed by the weight (kg) to height (m)^2^ ratio. Age, job, educational status, habitat (town or village) and physical activity level were recorded. Physical activity was assessed by the IPAQ and categorized as mild, moderate and severe.

### Assessment of semen parameters

Semen sampling was performed with 3 to 5 abstinence days. The use of lubricants was prohibited due to the spermicide properties. The sample was collected in sterile, plastic, and disposable collection vessels. After the liquefaction of samples in an incubator at 37 °C for 30 to 45 min, the macroscopic parameters, including volume, appearance, viscosity, and pH, were measured. Microscopic evaluation, including sperm count, morphology, vitality, and motility, was measured and interpreted according to the sixth edition of the WHO laboratory manual for the examination and processing of human semen [18]. The motility of sperm was categorized as progressive (slow and quick motility), nonprogressive, and immotile sperm.

Rapid and slow progressive sperm movement is defined as spermatozoa moving actively, either linearly or in a large circle, covering a distance, from the starting point to the end point, of at least 25 μm/s (for rapid) or 5 to < 25 μm/s (for slow). Nonprogressive is defined as spermatozoa with all other patterns of active tail movements with an absence of progression, and immotile is defined as spermatozoa with no active tail movements.

Sperm counting was performed using an improved Neubauer hemocytometer. In brief, semen samples were mixed properly, both chambers of the hemocytometer with 10 µl of semen were loaded, and the hemocytometer was examined with phase contrast optics at ×200 magnification to count at least 200 spermatozoa in each replicate.

For sperm morphology analysis, a smear of the semen sample was prepared by spreading 5–10 µl of semen sample on a microscope slide and allowing the slides to dry and then fixed with 95% (v/v) ethanol. The air-dried semen smear was stained by the Papanicolaou staining method. Briefly, the slides were sequentially immersed in ethanol, Harris’s hematoxylin, acidic ethanol, ethanol (50%, 80%), G-6 orange stain, ethanol, EA-50 green stain, and ethanol (95%, 100%). At least 200 spermatozoa per slide (two replicates) were evaluated using bright field optics at ×1000 magnification.

The vitality test using eosin–nigrosine can discriminate between dead sperm and immotile live spermatozoa. After mixing the semen sample, 50 µl of semen was mixed with an equal volume of eosin–nigrosin suspension. Thirty seconds later, a smear on a glass slide was prepared; then, the number of red- or pink-stained sperm (dead) or unstained white sperm (live) was counted (at least 200 spermatozoa) with a laboratory counter under bright field optics at ×1000 magnification and oil immersion. Then, the percentage of live cells was calculated.

The sperm DNA fragmentation assay kit (SDFA kit, Ideh Varzan Farda, Iran) was used to calculate the DNA fragmentation index (DFI). Briefly, 50 µl (5–10 × 106 sperm/ml) of washed semen sample with PBS was mixed with melted agarose, and then 20 µl of the cell suspension was placed on a coated glass slide, covered with a coverslip and incubated at 2–8 °C for 5 min. Afterwards, the coverslip was removed, and the slides were immersed in denaturing solution, incubated in the dark for 7 min, immersed in lysis solution for 15 min, washed with distilled water, dehydrated with alcohol and stained. The slides were observed under a light microscope at 1000× magnification, and at least 400 spermatozoa were scored.

DFI was classified based on the halo size of sperm cells, which was calculated as DFI (%) = (fragmented spermatozoa (spermatozoa with a small or no halo)/total number of spermatozoa counted) ×100. A DFI cutoff value less than 15% discriminated normal from borderline or abnormal sperm DNA status.

A sperm chromatin maturation assay kit (SCMA kit, Ideh Varzan Farda., Iran) was used to measure the sperm chromatin compaction status and abnormalities linked to nucleoprotein composition, which distinguished between lysine-rich histones and protamine-rich sperm nuclei. Briefly, a thin smear was prepared from a semen sample with a concentration of 10–15 million sperm/mL. After drying, the slides were processed with 3% buffered glutaraldehyde and then stained with aniline blue. A minimum of 400 sperm were checked using a light microscope. The percentages of less than 20% immature sperm histone-rich (the blue stain) to mature protamine-rich nuclei were considered normal.

### Dietary intake, GI and GL calculation

Dietary intake was determined by a validated 168-item questionnaire [19] that contains common dietary intake during the past 12 months (number of daily, weekly, monthly and annual). Data were inserted into the N4 software and converted to grams per day. The calculation of dietary GI and GL has been described previously [20]. We used published GI values that have been previously collected in a database [21]. Foods from the FFQ were matched to foods with reported GI values based on calorie and nutrient content, types of ingredients, and processing. For other foods, the GI was measured using standard methods. Dietary GI was calculated using the formula dietary GI = ∑foods C × F × GI ∕  ∑foods C × F, where C represents the grams of carbohydrate in a serving of food, F the frequency of consumption of the food, and GI the glycemic index using glucose as the reference. Dietary GL was calculated as dietary GL = ∑foods C × F × GI ∕ 100 or equivalently the product of total carbohydrate and dietary GI expressed as a percentage. The nutrients, dietary GI, and dietary GL were energy-adjusted using the residuals method [22].

### Sample size and statistical analysis

Considering power 0f 80% and α = 0.05, sample size was determined based on the previous study [25], through the below formula in which *P* = 0.4, d = 0.05; n=${{{z_{1 - \frac{\alpha }{2}}} \times {\text{p}}(1 - {\text{p}})} \mathord{\left/{\vphantom {{{z_{1 - \frac{\alpha }{2}}} \times {\text{p}}(1 - {\text{p}})} {{d^2}}}} \right.\kern-\nulldelimiterspace} {{d^2}}}$. By considering 10% dropout, 400 men were participated.

Data were analyzed using descriptive and analytical statistical tests using one variable and multivariable statistical test by analytical SPSS software, version 22. Data were checked by the Kolmogorov‒Smirnov test for normal distribution. To compare the assessed sperm parameters among the different quartiles of dietary GI and GL, one-way ANOVA test was used which was followed by post-hoc Tukey to determine the difference between each pair of means. A linear regression model was used to adjust the baseline parameters on outcomes. *P* < 0.05 was considered significant.

## Results

Of 400 men who met the inclusion criteria, only 322 men were finally analyzed. Seventy-eight men (20.4%) were smokers and were excluded from the final analysis. The mean age of the participants was 35.1 ± 5.9 yrs. The mean weight and BMI of the participants were 80.3 ± 10.1 kg and 26.6 ± 3.3 kg/m2, respectively. The mean intake of daily energy was 3067.2 ± 1525.8 kcal, protein was 100.8 ± 54.7 gr, fat was 108.2 ± 66.49 gr, and carbohydrate was 445.9 ± 242.2 gr. Thirty-six (9.4%) of the participants were illiterate. Sixty-one (61%) of the participants had a diploma, and one hundred twenty-nine men (33.8%) had a diploma. One hundred fifty-six participants (40.8%) had a university education. Nearly half of the participants (46.6%) had moderate levels of daily physical activity. Seventy-nine (20.7%) and one hundred twenty-five (32.7%) participants had mild and severe physical activity, respectively. The results of seminal fluid analysis, dietary GI and GL are illustrated in Table 1. As shown, the mean percentages of sperm vitality and DFI were lower than the normal values. Sperm with progressive and nonprogressive motility were at the lowest normal range compared to the reference ranges. The sperm DFI was moderate. Dietary GI and GL were higher than normal ranges in comparison to the reference values.

**Table 1:** Sperm-quality parameters, dietary glycemic index and load in the participants

Variables	Minimum	Maximum	Mean ± SD	Reference range [18, 23, 24]
Progressive motility				
quick slow	0.00 1	50 60	23.5 ± 16.2 30.4 ± 9.4	> 30%
Nonprogressive motility	10	58	30.9 ± 10.2	< 60%
Immotile	0.1	38	15.4 ± 10	< 10%
Count, mill/ml	0.00	9	27 ± 1.7	> 16
Normal morphology, %	1	9	5.3 ± 2.1	> 4%
Vitality, %	8	97	53.3 ± 29.8	> 54%
DFI, %	8	43	18.1 ± 7.6	Normal < 15% Moderate 15–30% Abnormal > 30%
SCMA, %	7	50	21.9 ± 9.3	Excellent < 20% Good: 20–30% Poor > 30%
Glycemic index	-	385.4	68.5 ± 44.8	Low:<55 Moderate: 56–69 High > 70
Glycemic load	-	252.8	28.4 ± 24	Low:<10 Moderate: 11–19 High > 20

DFI: DNA fragmentation index, SCMA: sperm chromatin maturation assay

Sperm-quality parameters were compared among quartiles of dietary GI in participants, and the results are shown in Table 2.

**Table 2:** Sperm-quality parameters in the highest quartile of dietary GI compared to the lowest quartile

Variables	Quartiles	Number	Means ± SDs	p value^†^
Progressive motility, %	1 2 3 4	94 99 94 95	59.6 ± 22.7 57.7 ± 27.4 47.5 ± 26.8 50.4 ± 22	< 0.001
Nonprogressive motility,%	1 2 3 4	94 99 94 95	28.7 ± 8.3 28.1 ± 10.8 35 ± 10 30.9 ± 10.2	< 0.001
Immotile, %	1 2 3 4	94 99 94 95	12.2 ± 7.8 11.3 ± 7.7 18.4 ± 10.8 15.4 ± 10	< 0.001
Count, mill/ml	1 2 3 4	94 99 94 95	30.2 ± 13.7 31.5 ± 22.2 18.2 ± 11.8 26.5 ± 14.5	< 0.001
Normal morphology, %	1 2 3 4	91 89 94 95	5.66 ± 2.2 5.8 ± 2.1 4.5 ± 1.7 5.4 ± 2.2	< 0.001
Vitality, %	1 2 3 4	90 95 87 95	45.2 ± 28.5 46.2 ± 29.5 64.4 ± 26.6 57.7 ± 30.3	< 0.001
DFI, %	1 2 3 4	94 99 94 95	16 ± 5.4 17.1 ± 8.2 19.3 ± 9.1 20.1 ± 8.9	< 0.001
SCMA, %	1 2 3 4	94 99 94 95	23.9 ± 9.2 20.6 ± 5.8 19.9 ± 9.1 23.03 ± 11.7	0.007

^†^Analyzed by one−way ANOVA among different quartiles. DFI: DNA fragmentation index, SCMA: sperm chromatin maturation assay

Post hoc analysis showed that men in the 3rd and 4th quartiles of dietary GI had lower progressive sperm than those in the first quartile (*p* < 0.001 and *p* = 0.002). Participants in the first and second quartiles of dietary GI had significantly lower nonprogressive sperm than those in the 3rd and 4th quartiles (*p* < 0.001, in all comparisons). Men in the 3rd and 4th quartiles of dietary GI had significantly higher immotile sperm than those in the first quartile (*p* < 0.001, in both). The sperm count was significantly different among the different quartiles (*p* < 0.001). The sperm count was significantly lower in the 3rd quartile than in the first quartile (*p* < 0.001). The percentages of sperm with normal morphology significantly decreased in the 3rd quartile compared to the first and 2nd quartiles (*p* < 0.001 in both). Sperm vitality significantly decreased in participants in the 3rd quartile compared to the first and 2nd quartiles (*p* < 0.001 and *p* = 0.001, respectively). The DFI significantly increased in the 3rd and 4th quartiles compared with the first quartile (*p* = 0.01 and *p* = 0.001, respectively); however, SCMA significantly decreased in the 3rd quartile compared to the first quartile (*p* = 0.01).

**Table 3:** Sperm-quality parameters in the highest quartile of dietary GL compared to the lowest quartile

Variables	Quartiles	Number	Means ± SDs	p value^†^
Progressive motility, %	1 2 3 4	95 95 96 95	69.03 ± 14 60.3 ± 25.4 47.1 ± 10.4 37.7 ± 6.4	< 0.001
Nonprogressive motility,%	1 2 3 4	95 95 96 95	24.4 ± 5.06 26.7 ± 8.5 33.5 ± 10.2 38.9 ± 10.2	< 0.001
Immotile, %	1 2 3 4	95 95 96 95	7.7 ± 5.01 11.3 ± 9 17.7 ± 8.9 25.2 ± 6.3	< 0.001
Count, mill/ml	1 2 3 4	95 95 96 95	38 ± 19.9 26.1 ± 18.6 20.8 ± 10.9 22 ± 10.8	< 0.001
Normal morphology, %	1 2 3 4	87 91 95 95	6.6 ± 1.9 6.2 ± 2.1 4.8 ± 1.9 3.8 ± 1.2	< 0.001
Vitality, %	1 2 3 4	89 88 94 95	27.5 ± 14.4 39.8 ± 26.4 62.1 ± 29.3 81.3 ± 9.6	< 0.001
DFI, %	1 2 3 4	95 95 96 95	13.5 ± 3.06 15.8 ± 6.2 20.6 ± 7.4 23.4 ± 8.07	< 0.001
SCMA, %	1 2 3 4	95 95 96 95	25.7 ± 7.4 24.7 ± 9.4 21.5 ± 10.4 15.4 ± 5.5	< 0.001

^†^Analyzed by one−way ANOVA among different quartiles. DFI: DNA fragmentation index, SCMA: sperm chromatin maturation assay

Post hoc analysis on sperm-quality parameters among different quartiles of dietary GL showed that there was a significant difference in sperm with progressive motility in men in the 2nd, 3rd and 4th quartiles compared to the first quartile (*p* < 0.001 in all). The lowest progressive property was shown in the last quartile compared to the others (*p* < 0.001 in all). Men in the highest quartile of dietary GL had higher nonprogressive sperm than those in the 3rd and 1st quartiles (*p* < 0.001 in all). The percentages of immotile sperm were significantly associated with a higher dietary GL (*p* = 0.006 in the 2nd group, *p* < 0.001 in the 3rd group, and *p* < 0.001 in the 4th group) compared to the first quartile. The sperm count was negatively associated with an increase in dietary GL quartile. A significant decrease was shown between the 2nd, 3rd and 4th quartiles compared with the first quartile (*p* < 0.001 in all). The percentages of sperm with normal morphology showed a significant negative association with the 3rd and 4th quartiles of dietary GL compared to the first quartile (*p* < 0.001 in both). Sperm vitality showed a significant positive association with dietary GL (*p* < 0.001 in all). The lowest vitality was shown in the first quartile of dietary GL compared to the others (*p* < 0.001 in all). Sperm DFI showed a significant positive association with the 3rd and 4th quartiles of dietary GL compared to the first quartile (*p* < 0.001 in both). Moreover, sperm DFI was significantly higher in the 2nd quartile than in the 3rd and 4th quartiles (*p* = 0.02 and *p* < 0.001, respectively). SCMA was significantly higher in the first quartile of dietary GL than in the 3rd and 4th quartiles (*p* = 0.003 and *p* < 0.001, respectively). SCMA was significantly at the lowest level in the 4th quartile compared with the others (*p* < 0.001 in all). (Table 3).

Adjusting for the baseline parameters, the association between dietary GI and GL was assessed with sperm motility. Dietary GI showed a positive association with sperm progressive motility (OR = 0.14, 95% CI: 0.15 − 0.1, *p* = 0.01); however, dietary GL showed a negative association (OR=-0.47, 95% CI: -0.56, -0.39, *p* < 0.001). Unlike sperm progressive motility, GI showed a negative association with sperm nonprogressive properties (OR=-0.32, 95% CI: -0.1, -0.45, *p* < 0.001); however, dietary GL showed a positive association with this property (OR = 0.58, 95% CI: 0.2–0.3, *p* < 0.001). Higher dietary GI was associated with lower immotile sperm (OR= -0.11, 95% CI: -0.05, -0.001, *p* = 0.03); however, higher dietary GL was associated with higher levels (OR = 0.4, 95% CI: 0.2–0.29, *p* < 0.001). A higher dietary GI was associated with a higher sperm count (OR = 0.23, 95% CI: 0.004–0.01, *p* = 0.001). In contrast, higher dietary GL was associated with a lower sperm count (OR = 0.35, 95% CI:-0.03, -0.02, *p* < 0.001). Sperm percentages with normal morphology were associated with a higher dietary GI by 70% (95% CI: 0.008–0.02, *p* < 0.001). However, higher dietary GL was associated with lower percentages of sperm with normal morphology by 40% (95% CI: -0.06, -0.04, *p* < 0.001). Dietary GI (OR=-0.16, 95% CI: -0.05, -0.006, *p* = 0.01) and GL (OR = 0.49, 95% CI: 0.12–0.19, *p* < 0.001) levels showed a significant association with sperm DFI. Dietary GI (OR = 0.16, 95% CI: 0.008–0.06, *p* = 0.01) and GL (OR=-0.44, 95% CI:-0.22, -0.12, *p* < 0.001) showed a significant association with SCMA.

## Discussion

The association between dietary GI and GL was assessed in men who attended the infertility clinic of Zanjan University of Medical Sciences, Zanjan, as a mountainous city in northwestern of Iran with a Turkish ethnicity. Dietary GI showed a negative association with sperm motility, vitality, morphology and DFI, but showed a positive association with SCMA. Dietary GL showed a negative association with sperm motility, count, morphology, DFI and SCMA. However, higher dietary GL showed a positive association with sperm vitality. Adjusting for the baseline parameters, higher dietary GI was associated with a 77% higher sperm count. In contrast, higher dietary GL was associated with a 65% lower count. In fact, it can be suggested that nutritional recommendations be individualized. Men who have problems with sperm viability and SCMA may benefit from a diet with a higher GI and GL level; however, a diet with lower GI and GL may be recommended for men with complications in sperm motility, count and DFI. Dietary habits and preferences for local natural foods create differences in dietary patterns in this area. Moreover, people consume more olives, fish and olive oils along with animal fats. These differences in dietary habits justify the need to conduct studies on the relationship between diet and chronic diseases in each city. On the other hand, Zanjan is an industrial city with lead and zinc mines, which affect the water and soil of the region. This contamination affects the fertility status of men and women living in this area.

The GI is defined as the area under the 2 h curve of postprandial glucose after the consumption of a food product containing 50 g of digestible carbohydrate and expressed as the ratio of the glycemic response to the same amount of reference carbohydrate from glucose or white wheat bread consumed by the same person [26]. Integrating the GI of the food with the amount of given carbohydrates in a portion size is named the GL, which provides a more accurate picture of postprandial glycemia [27]. Higher dietary GI and GL are related to hyperinsulinemia and related disorders, including obesity, type 2 diabetes, cardiovascular diseases, and some cancers [28, 29]. However, the sperm-quality parameters, DNA fragmentation and chromatin maturation of sperm have not been studied. Recently, the relationship between the level of reproductive hormones and the quality of semen in diabetic patients was investigated in men who live in Iraq, and the results showed that the count, motility, and normal morphology of sperm are affected by hyperglycemia [30]. In our study, participants had no metabolic complications, including hyperglycemia, hyperlipidemia, hypertension, and thyroid disorders. The design of the present study was cross-sectional, while the mentioned study was conducted as a case-control study between diabetic and nondiabetic subjects. Moreover, the diet of the participants was not examined, and only the blood sugar level and sperm quality parameters were examined in a small sample size. In another recent study conducted in Denmark, the effect of sweetened drinks was compared with that of artificial sweeteners on the quality of semen in adolescents, which did not reach a significant difference between the two groups. However, only the normal morphology was affected in adolescents who consumed artificially sweetened beverages more than 3 days per week [33]. Two other studies that were recently conducted in Israel and Spain showed a negative relationship between dietary sugar consumption and sperm parameters in a dose-dependent manner [31, 32]. One of the proposed mechanisms is related to hyperinsulinemia induced by hyperglycemia which leads to impairment of sperm glucose uptake, metabolism and glycolysis pathway [33, 34]. Other mechanism related to the inhibition of aromatase, a converting enzyme of testosterone to estradiol, by high dietary GI and GL which lead to low sperm production due to oxidative stress and mitochondrial dysfunction in germ-producing Leydig cells [35, 36]. Germ cells and mature spermatozoa are susceptible to oxidative stress, which decrease sperm quality and quantity [37].

A recent meta-analysis study reported that obesity was associated with a decrease in seminal fluid volume (mean difference: -0.25, 95% CI: -0.33 to -0.16), sperm count (mean difference: -23.84, 95% CI: -30.36 to -17.33), concentration (mean difference: -7.26, 95% CI: -10.07 to -4.46), progressive mobility (mean difference: -5.68, 95% CI: -8.79 to -2.56), and serum testosterone level (mean difference: -1.11, 95% CI: -1.92 to -0.30) [38]. The results of our study showed that weight only had a significant effect on the progressive movements of sperm (OR = 0.09, 95% CI: 0.01–0.338), and a negative effect near the significance level on sperm vitality that may become significant with an increase in sample size. In this meta-analysis, diet was not evaluated, and only the correlation between BMI and sperm parameters was assessed. Another study that was recently conducted in Iran investigated sperm parameters, DNA integrity and protamine expression in patients with type 2 diabetes in a small sample size study. The results showed that diabetes and hyperglycemia not only decrease the quality of sperm parameters but also affect the sperm maturation process by increasing the significant consequences at the DNA/chromatin levels in diabetic patients [36]. In this study, in addition to the different sample sizes and study designs, dietary GI and GL were not investigated. In general, all studies have compared the parameters of semen in diabetic patients with healthy individuals [39–41], and there is no study on the relationship between dietary glycemic index and load in men with sperm-quality parameters, DNA fragmentation and chromatin maturation of sperm in people who do not have any metabolic disorder. Only one cross-sectional study has examined the relationship between micronutrient consumption and semen quality. This study found that an increase in saturated fatty acids and a decrease in linoleic acid and selenium intake were related to sperm count. Decreases in dietary intake of selenium, zinc and vitamin E were also associated with sperm concentration. In addition, zinc, vitamin E, selenium and vitamin D had a significant relationship with sperm motility. The intake of saturated fatty acids, linoleic acid, and zinc was related to sperm morphology. A significant adverse relationship was found between sperm quality and increased intake of saturated fatty acids from the diet and decreased intake of linoleic acids, selenium, zinc, vitamin E and vitamin D [41]. Micronutrients are not consumed individually and are consumed in a dietary pattern. Therefore, recommending changes in dietary patterns is easier and more applicable than micronutrient changes.

In summary, the present study showed that men who live in Zanjan City consume a diet with moderate GI and high GL, which can affect the quantity, quality and maturation of sperm and ultimately male infertility. One of the advantages of the present study was the large sample size. All the participants in the present study were healthy and did not have any chronic disorders, including hyperglycemia, hyperlipidemia, hypertension, etc., at least in the last 6 months and were not taking any medication, which increases the power of the study. Similar to all studies, the present study also had limitations. The study was cross-sectional, which means that it cannot establish causality between dietary GI and GL and sperm parameters. Moreover, the study only included men attending an IVF clinic, which may not be representative of the general population. The study relied on self-reported dietary intake, which may be subject to recall bias. Finally, the study was conducted in Iranian men who living in Zanjan city, so the results may not be generalizable to other populations. More studies are needed to assess the association between dietary GI and GL with sperm parameters in other populations due to the diversity in dietary intake and habits. Future studies could use a longitudinal design to establish causality between diet and sperm parameters. Additionally, studies could include a more diverse sample of men from different settings and geographic locations to improve generalizability. Finally, future studies could use objective measures of dietary intake, such as biomarkers or dietary records, to reduce the risk of recall bias. Future studies could include a more comprehensive assessment of fertility status in participants, such as semen analysis and reproductive hormone levels. Additionally, studies are proposed to investigate the effects of individual foods or food groups on sperm parameters to provide more targeted dietary recommendations for infertile men.

## Conclusions

The present study showed that men who live in Zanjan City consume a diet with moderate GI and high GL, which can affect the quantity, quality and maturation of sperm and ultimately male infertility. This issue should be considered in nutrition education programs to community. Moreover, policy- and decision-makers at the highest level of government to provide a solution.

## Data Availability

All the data relevant to the manuscript are reported in tables. The raw data can be accessed from the corresponding author upon request.
